# Non-inferiority of creatinine excretion rate to urinary L-FABP and NGAL as predictors of early renal allograft function

**DOI:** 10.1186/1471-2369-15-117

**Published:** 2014-07-16

**Authors:** Jernej Pajek, Andrej Škoberne, Klara Šosterič, Barbara Adlešič, Bojan Leskošek, Maja Bučar Pajek, Joško Osredkar, Jelka Lindič

**Affiliations:** 1 Department of Nephrology, University Medical Centre Ljubljana, Zaloška 2, 1525 Ljubljana, Slovenia; 2 Faculty of Medicine, University of Ljubljana, Ljubljana, Slovenia; 3 Faculty of Sport, University of Ljubljana, Ljubljana, Slovenia; 4 Institute of Clinical Chemistry and Biochemistry, University Medical Centre Ljubljana, Ljubljana, Slovenia

**Keywords:** Biomarker, Kidney transplantation, Delayed graft function, Outcome

## Abstract

**Background:**

We evaluated accuracy of urinary liver type fatty acid-binding protein (L-FABP) for prediction of early allograft function and compared it to neutrophil gelatinase associated lipocalin (NGAL), diuresis and urinary creatinine excretion rate (UCr).

**Methods:**

Urine samples from 71 consecutive patients were taken 4, 10, 24 and 48 h after transplantation. We classified recipients into two groups: immediate graft function (IGF), with more than 70% reduction of serum Cr at 7th day post-transplant, and delayed graft function (DGF)/slow graft function (SGF) group (DGF - the need for hemodialysis procedure in the first week, SGF - less than 70% reduction of serum Cr in the first week).

**Results:**

Thirty-one recipients had IGF and 40 had DGF/SGF. L-FABP was only useful 48 h post-transplant with ROC AUC of 0.85 (95% C.I. 0.74-0.92); NGAL 24 h post-transplant had ROC AUC of 0.82 (0.7-0.91). Sensitivity, specificity, PPV and NPV for prediction of DGF/SGF with L-FABP > 9.5 mg/mmol Cr and NGAL > 33.1 μg/mmol Cr were: 86, 80, 83 and 83% (L-FABP), and 68, 93, 91, and 73% (NGAL). The difference in urine output between the groups was largest 4 h post-transplant (p = 0.001), later on the difference diminished. There were no significant differences in ROC AUC between L-FABP at 48 h, NGAL at 24 h, urine output at 4 h and UCr excretion rate at 10 h post-transplant. UCr < 0.56 mmol/h 10 h post-transplant predicted DGF/SGF with 94% sensitivity, 84% specificity, 89% PPV and 91% NPV, ROC AUC was 0.9. Classification tree with urine output 4 h and UCr 10 h post-transplant accurately predicted 89% of outcomes. When L-FABP or NGAL were added, the prediction was accurate in 92 or 90%, respectively.

**Conclusions:**

L-FABP is comparable to NGAL for prediction of first week allograft function, however UCr and diuresis were non-inferior.

## Background

Acute kidney injury (AKI) that occurs at transplantation often progresses to delayed graft function (DGF) [1]. DGF may be defined as an allograft injury that causes the use of dialysis in the first week after transplantation [2]. It is associated with increased risk of graft loss, acute rejection and worse allograft function [3]. While it is possible to predict the risk of DGF through the use of many donor- and recipient-associated factors [4], the prediction is not perfect and markers with a better predictive value are warranted.

In the recent years, the urine biomarkers of AKI have become available. Neutrophil gelatinase-associated lipocalin (NGAL) is a protein from lipocalin family and induced in the renal tissue in the conditions of ischemia-reperfusion injury, with tissue protective properties [5]. In the studies examining its predictive capability for DGF area under the receiver operating characteristic (ROC) curve was between 0.75 and 0.9 [6-8], thus showing a good prognostic value for DGF. Similar were results for urine interleukin-18 (6, 7). Another interesting marker of ischemia-reperfusion injury is liver type fatty acid binding protein (L-FABP) found in proximal tubular cells. During ischemic injury L-FABP binds lipid peroxidation products and alleviates oxidative stress [9]. It is useful for diagnosis of various forms of AKI [10] and its urinary concentration showed an excellent correlation with ischemia time in living-related kidney transplantation [9]. Its possible role as a marker and predictor of allograft function in the clinical setting of deceased donor kidney transplantation (DDKT) has been examined [11] but no data on exact predictive value for early allograft function were reported so far.

When looking at studies of the predictive values of various urinary biomarkers for early allograft function there are several uncertainties left unanswered. It is long known that the level of diuresis after kidney transplantation is useful and perhaps one of the most clinically used predictors of allograft function, however in many of the studies the predictive value of urinary biomarkers was not rigorously compared to the level of diuresis. Often, an arbitrary level of urine output of 1 liter on day 1 post transplant was used to compare the predictive value of diuresis to various urinary biomarkers [7,8], however the predictive value of diuresis after transplant may vary with time and clinical protocols of fluid and diuretic management. Furthermore, urinary creatinine (UCr) concentration, another traditionally used predictor of kidney function recovery [12], was often not reported in these studies. Currently therefore it is hard to answer what is the exact benefit of novel urinary biomarkers above and beyond the traditionally used (and very cheap) monitoring of diuresis and UCr. Finally, the normalization of urinary biomarker to UCr concentration was variable between studies and it is not clear if there is a consistent benefit of normalization to UCr concentration for increasing the predictive value of urine biomarkers.

We have designed this study with two main goals. First, we wanted to examine the prognostic accuracy of urinary L-FABP for early allograft function in deceased donor kidney transplantation and to compare it to the well established urinary biomarker NGAL. We searched for the best timing of L-FABP sampling and analyzed the impact of normalization to UCr. Second, we used this data to compare the value of both novel urinary biomarkers to traditional predictors of allograft recovery - diuresis and UCr concentration. The product of both quantities - UCr excretion rate - was also analyzed in this regard.

## Methods

We conducted a single centre prospective observational cohort study of adult patients receiving a deceased-donor kidney transplant in the period from November 1st, 2010 till May 28th, 2012. We obtained informed consent from all participants and the research was approved by the national medical ethics committee. All transplantations were conducted through Eurotransplant program. The recipients of DDKT were excluded from the study in case of early surgical complication known at time of biomarker sampling (renal vein and arterial trombosis) and transplantectomy. Induction immunosuppression routinely consisted of basiliximab, cyclosporine, mycophenolate mofetil and corticosteroids [13].

Patient and donor data were obtained from interview, patient medical documentation and Eurotransplant donor medical files before the transplant operation. Time of transplantation was defined at the time of surgical establishment of blood flow through the allograft in the recipient. After this time point the urine samples were taken 4, 10, 24 and 48 h later (urine samples 1,2,3 and 4). These samples were taken from the fresh urine obtained with bladder catheter in all cases. A minimum of 20 ml of urine was sampled and immediately transported to laboratory for centrifugation and storage. In a subset of patients with residual diuresis voided urine sample before transplantation was obtained (urine sample 0). At the time of collection of post-transplant samples hourly diuresis was measured with the routine clinical urine collecting bag and measurement container to the nearest 5 ml.

Urine was centrifuged 10 minutes at 3000 rpm and supernatant was stored at -30°C. Urine creatinine (UCr) concentration was measured spectrophotometrically with the use of standard kinetic Jaffe’s reaction (system Dimensionâ Xpand, Siemens). Serum creatinine (SCr) concentrations were measured with the kinetically compensated Jaffe’s method (system Advia 1800, Siemens, Tarrytown, USA). UCr excretion rate at the sampling time points in mmol/h was calculated by multiplying UCr concentration of the sample with the hourly diuresis in the last hour before the sampling. NGAL concentration was measured only in the sample 3 (24 h post-transplant). Turbidimetric immune assay (Bioporto Diagnostics) on the system Olympus AU 400 was used. All samples were measured in the same analytic procedure, intra-assay variability was up to 6%. L-FABP concentration was measured in the samples 0 to 4. Enzyme linked immunoassay was used (HK404 Human L-FABP set, Hycult Biotech). All samples were measured in the same series with intra-assay variation of 9.5%.

SCr concentration was routinely measured 3-5 hours after transplant operation and at each post-operative day in the first week. When the relative SCr decrement was more than 70% in the first post-transplant week, the recipients were designated as immediate graft function (IGF). Relative SCr decrement was calculated as the difference between first SCr value at 3-5 h on day 0 and the value on day 7 divided by Scr at 3-5 h on day 0. Recipients who had a relative SCr decrement lower than 70% were designated as slow graft function (SGF) and recipients with at least one haemodialysis procedure in the first post-transplant week were designated as delayed graft function (DGF). For the purpose of statistical analysis the recipients were divided into two groups: IGF and others (SGF and DGF combined). All statistical tests were performed two-tailed with a significance level of 0.05. Results are presented as mean ± SD for normally distributed parameters, median (range) for non-normally distributed ones and number of recipients (percent) for nominal variables.

Differences between both groups were analyzed using *T*-test, Mann-Whitney’s *U* test and chi-squared test as appropriate. We performed ROC curves to test the discriminating value of markers for the first post-transplant week allograft function (either IGF or SGF/DGF). ROC curve is a graphical plot which illustrates the performance of a binary classifier system as its discrimination threshold is varied. It is created by plotting sensitivity (true positive rate) against 1-specificity (false positive rate) at various threshold settings. Youden’s index (the point on ROC curve with maximal value of sensitivity and specificity sum) was used to define the optimal cut-off point for each marker.

We have made a determination of the sample size under the assumption of area under the ROC curve of a putative biomarker of 0.8, with alpha type mistake of 0.05 and beta of 0.1. Under this assumptions the number of subjects needed to reach statistical significance for this AUC was 28 in the positive and 28 in the negative category - 56 total.

Classification tree method with binary algorithm was used to test the added predictive value of L-FABP or NGAL when diuresis and creatinine excretion rate were also used. In the next step testing of each constructed classification tree was performed with the “leave-one-out” method. Here, each time a single recipient is excluded from the classification tree construction and then the constructed tree is tested for correct prediction of the excluded recipient. This way, the percentage of correct predictions can be calculated and compared with different markers used in the classification tree. For statistical comparison of areas under ROC curves Medcalc application was used (v.12.3., Medcalc software, USA); for all other statistical analyses IBM SPSS Statistics software was used (IBM, USA).

## Results

**Figure 1:**
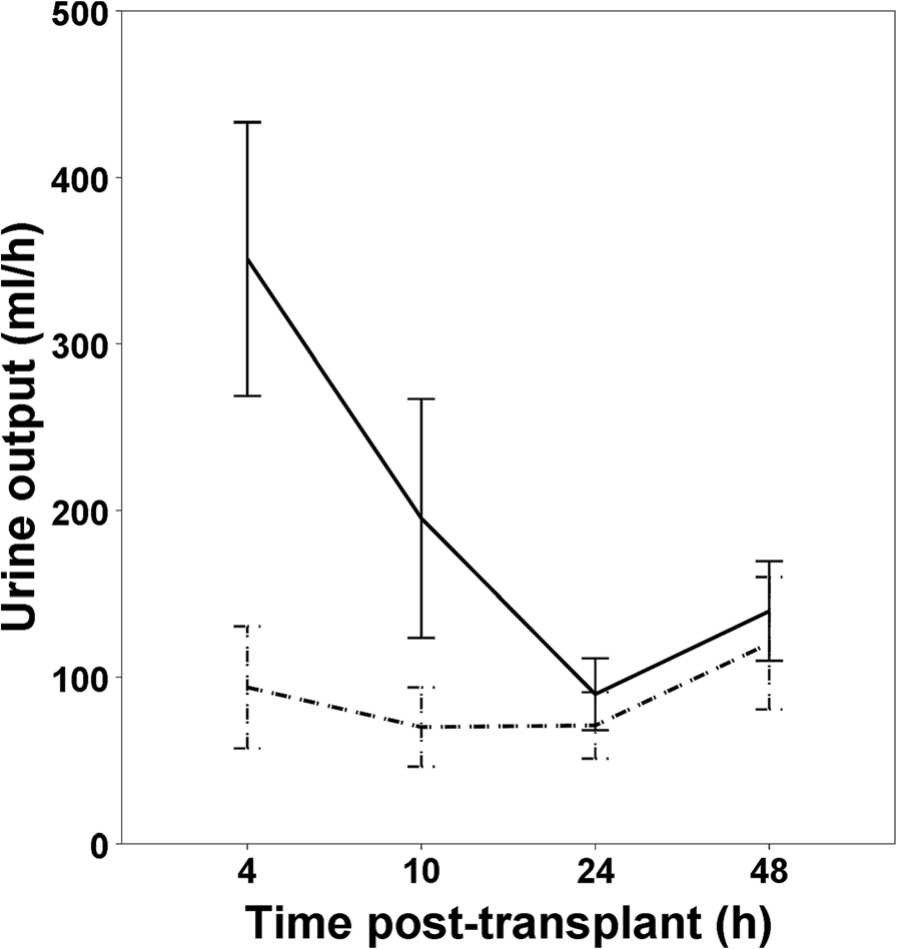
**Urine output in the first 48 h after transplantation.** Solid line - IGF group. Dashed line - SGF/DGF group. Bars represent 95% C.I.

In the study period 74 DDKT recipients were included. Three recipients were excluded from the analysis due to complications in the first days known at time of urine sampling (3 renal vein thromboses). Mean age of 71 remaining recipients was 50 ± 12 years, 56% were male. Median residual urine output was 400 ml per day (0-3000 ml), 27 recipients were anuric (100 ml urine daily or less). There were 31 recipients (44%) with IGF, 20 (28%) with SGF and 20 (28%) with DGF. SGF and DGF patients were combined in the single SGF/DGF group of 40 patients (56%). Clinical parameters influencing early graft function are shown in the Table 1.We tested urine output, UCr excretion rate, NGAL and L-FABP as predictive markers of early allograft function in the first post-transplant week. Urine output at 4 sampling time points after transplantation is shown in Figure 1. The difference between IGF and SGF/DGF groups was largest immediately after operation and decreased later on. Therefore we used urine output 4 h after transplantation for analysis of diuresis as predictive marker.

**Table 1:** Clinical parameters influencing early graft function^a^

**Parameter**	**All recipients (n = 71)**	**IGF (n = 31)**	**SGF and DGF (n = 40)**	**p**
Residual urine output (ml/day)	400 (0-3000)	500 (0-3000)	200 (0-2500)	0.04
Marginal donor number (N (%))	20 (28%)	8 (26%)	12 (30%)	0.79
Donor age (years)	48 ± 12	44 ± 14	51 ± 9.5	0.02
Preterminal donor serum creatinine (μmol/l)	86 ± 40	85 ± 40	86 ± 40	0.87
Donor acute kidney injury (N (%))^b^	13 (18%)	5 (16%)	8 (20%)	0.76
Cold ischemia time (hours)^c^	17.5 ± 5.6	15.5 ± 4.5	19 ± 5.8	0.006
Anastomosis time (minutes)^d^	47 ± 17	44 ± 16	49 ± 17	0.21
Donor kidney length (cm)	11.5 ± 1	11.8 ± 1.1	11.3 ± 0.8	0.04

^a^Results are presented as mean ± SD for normally distributed parameters, median (range) for non-normally distributed ones and number of recipients (percent) for nominal variables.

^b^Defined as pre-terminal rise of donor serum creatinine for 25% or more.

^c^Defined as the period from the start of cold perfusion of aorta in the donor till the establishment of blood flow through the allograft in recipient.

^d^Defined as the time period from removal of the allograft from the cold solution till the establishment of blood flow through the allograft in recipient.

**Figure 2:**
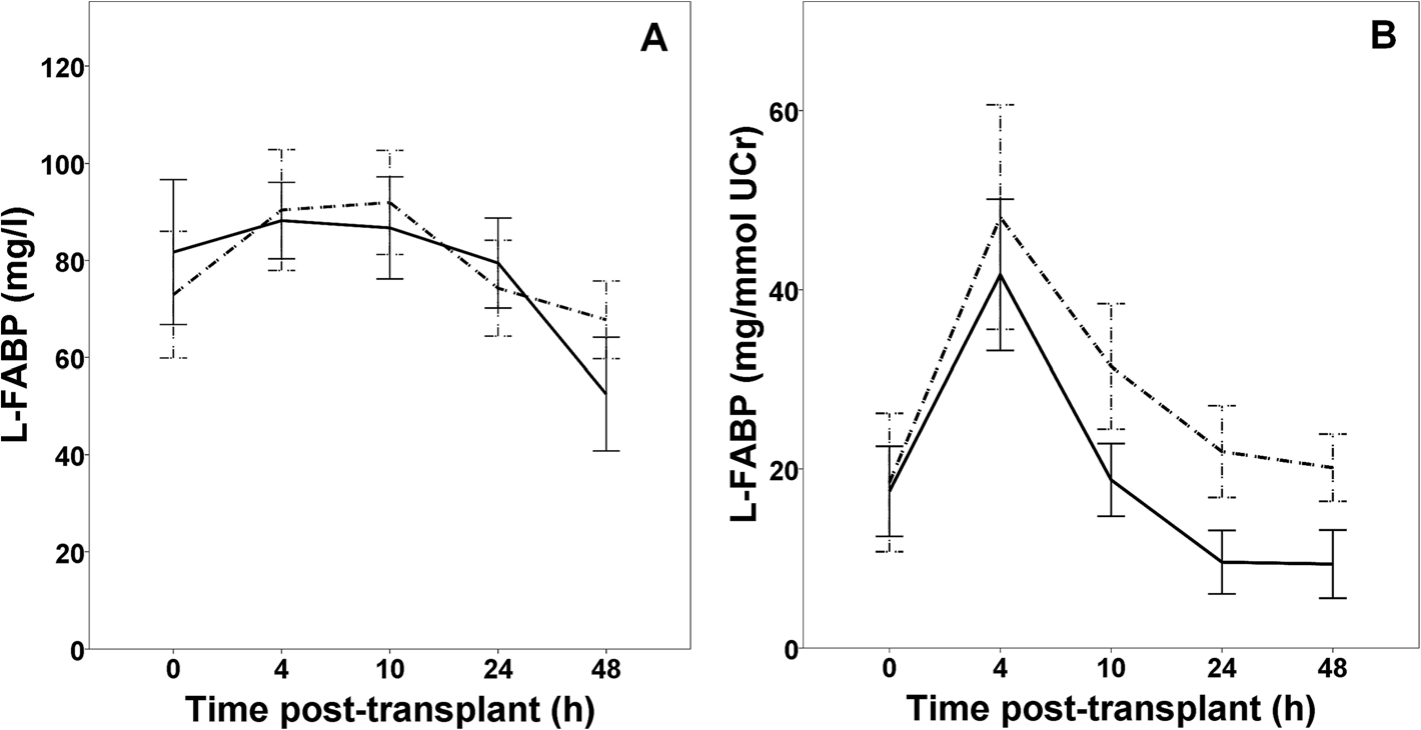
**Urinary L-FABP.** L-FABP urinary concentration **(A)** and L-FABP urinary concentration normalized to urinary creatinine - UCr **(B)** in the first 48 h after transplantation. Solid line - IGF group, dashed line - SGF/DGF group. Bars represent 95% C.I.

When L-FABP and NGAL were analyzed for discriminative and predictive value of allograft function, results were consistently improved if normalization of their urinary concentrations to UCr concentration was used. L-FABP urinary concentrations with time for both groups of recipients are shown in Figure 2 (2A for urinary concentrations and 2B for concentrations normalized to UCr). Significant difference between both groups for non-normalized L-FABP concentrations was only found at 48 hours after transplantation: urinary L-FABP was 55.1 (range: 0.6-127) and 69.5 (13.1-125.2) mg/L in IGF and SGF/DGF groups, respectively (p = 0.018). Area under ROC curve (ROC AUC) for L-FABP concentration at 48-hour was 0.65 (95% C.I. 0.53-0.77). When normalization to UCr was made, the ROC AUC was significantly larger, both for L-FABP and NGAL (p = 0.005 and 0.001, respectively). Urinary NGAL concentrations were 91.8 (3.5-381.8) and 274.9 (3.5-2390) μg/L in IGF and SGF/DGF groups respectively, p = 0.042, and ROC AUC was 0.69 (0.56-0.8). ROC AUC for urinary concentrations normalized to UCr, together with sensitivity, specificity and the median values for both groups are shown in the Table 2.

**Table 2:** Discriminant values of urine markers for allograft function

**Predictive marker**	**N**	**Time post-transplant (h)**	**IGF**	**SGF and DGF**	**p**^**a**^	**ROC AUC (95% C.I.)**^**b**^	**Optimal threshold value (sensitivity, specificity)**^**c**^
Urine output (ml/h)	67	4	390 (20-950)	50 (0-325)	<0.001	0.87 (0.77-0.94)	325 (100%, 57%)
UCr excretion rate (mmol/h)^d^	58	10	0.99 (0.02-2.18)	0.26 (0-0.72)	<0.001	0.9 (0.8-0.96)	0.56 (94%, 84%)
	67	24	0.85 (0.03-1.85)	0.25 (0-0.88)	<0.001	0.9 (0.8-0.96)	0.46 (78%, 94%)
L-FABP (mg/mmol UCr)	65	48	7.1 (0.1-53.5)	20 (4.7-75.2)	<0.001	0.85 (0.74-0.92)	9.5 (86%, 80%)
NGAL (μg/mmol UCr)	60	24	8.7 (0.2 – 107.1)	62.6 (1.8-770.9)	<0.001	0.82 (0.7-0.91)	33.1 (68%, 93%)

The discriminatory values of urine output, UCr excretion rate, L-FABP and NGAL are shown for early allograft function - the distinction between IGF and SGF/DGF groups.

^a^Mann-Whitney’s *U* test for difference between the groups.

^b^The differences between AUC for urine output, UCr at 10 h, L-FABP and NGAL were not statistically significant.

^c^for prediction of SGF/DGF; for urine output and UCr values below the optimal threshold value, for L-FABP and NGAL values above the threshold value.

^d^UCr - urinary creatinine.

UCr excretion rate at 10 hours post-transplant had the largest area under ROC curve. Since recipients with residual urine output could excrete significant amount of creatinine through their native kidneys we divided recipients into two groups according to residual diuresis (anuric recipients and others). For both groups similar and consistent differences in UCr excretion rate were found between IGF and SGF/DGF subgroups (exact results are shown in Additional file 1). The positive and negative predictive values for UCr excretion rate compared to other markers are shown in the Table 3.

**Table 3:** Positive and negative predictive value for slow or delayed graft function in the first post-transplant week

**Predictive marker**	**PPV**^**e **^**(%)**	**NPV**^**d **^**(%)**
^a^Urine output < 325 ml/h	74	100
^b^UCr excretion rate < 0.56 mmol/h	89	91
^c^UCr excretion rate < 0.46 mmol/h	93	78
^c^NGAL > 33.1 μg/mmol UCr	91	73
^d^L-FABP > 9.5 mg/mmol UCr	83	83

^a^4 h post-transplant; ^b^UCr - urinary creatinine excretion rate 10 h post-transplant; ^c^24 h post-transplant; ^d^48 h post-transplant. ^e^PPV, positive predictive value; ^d^NPV, negative predictive value. Estimated prevalence of slow and delayed graft function was 56%.

Added predictive value of L-FABP or NGAL above that of urine output and UCr excretion rate was analyzed using the method of classification trees. When using solely urine output and UCr excretion rate 89% of outcomes (63 out of 71) could be correctly predicted (the outcomes being IGF or SGF/DGF). When L-FABP normalized to UCr at 48 h post-transplant was added to classification tree, 92% of outcomes were correctly predicted (65 out of 71). When NGAL was used, 90% (64 out of 71) outcomes were correctly predicted. (The constructed classification trees are shown in Additional file 2). After the first week there were two patients with early rejections diagnosed. In a post-hoc analysis with these two patients excluded - a way to obtain a clearer patient sample with pure ischemia-reperfusion injury, the results were essentially unchanged (exact results not shown).

## Discussion

In the present study we measured urinary L-FABP, NGAL, diuresis and UCr to find the best predictive marker for early allograft function in deceased donor kidney transplantation. Finding a reliable biomarker for early allograft function has two major implications. First is the clinical use in cases of poor early allograft function. When differential diagnostic possibilities of competing pre-renal and post-renal diagnoses are excluded, patients with DGF should be biopsied to discriminate between the presence of acute rejection and acute tubular necrosis. The exact time point of the kidney biopsy is variable in this scenario. However, when a reliable biomarker would suggest a good recovery of kidney function but this would not be the case, the clinician could pursue an earlier diagnostic evaluation and treatment intervention. This was also the reason for dividing patients to the two outcome groups: patients with immediate graft function and others (SGF and DGF patients). A second important demand for pursue of optimal predictive biomarker in the early post-transplant period comes from the fact that several potential targets in ischemia-reperfusion mechanisms are already identified and new treatments for DGF are or will be tested to alleviate ischemia-reperfusion injury [1]. Having a good early biomarker of the ischemia-reperfusion injury would aid to identify the patients where an early intervention would be beneficial.

Our results show that the predictive value of urinary L-FABP for DGF and SGF is comparable to NGAL. The optimal time to sample urine for L-FABP was 48 h post transplant and the area under ROC curve was similar to that of NGAL. Good positive and negative predictive value for DGF and SGF were found (83% both). We have only sampled NGAL at 24 h post-transplant since this biomarker was extensively evaluated in previous studies [7,8] and this time point was found to be appropriate. This is a slight comparative advantage for NGAL since according to our data, best predictive value for L-FABP is only obtained 48 h post-transplant so NGAL allows earlier prediction of allograft function. So far, L-FABP has been studied in a living-donor kidney transplant cohort, where no value for prediction of early rejection could be found [14]. Przybylowski et al studied L-FABP in a cohort of heart and kidney allograft recipients and found that urinary L-FABP correlated with renal allograft function concluding that it could potentially serve as a marker for impaired kidney function [11]. Our study is to our knowledge the first report on the comparative value of L-FABP as a predictor of early allograft function in DDKT with comparison to a well established urinary marker NGAL and UCr.

We consistently found improvement in predictive ability when urinary L-FABP concentrations were normalized to UCr concentration. UCr excretion fluctuates in the conditions of unstable kidney function and this was shown to importantly influence urine biomarker to UCr ratio (12). Specific to the case of early post-transplant period is the finding that lower UCr concentration in SGF/DGF patients will tend to inflate biomarker to UCr ratio and higher UCr in IGF patients will lower this ratio. Therefore, as noted in a theoretical model, a poor renal allograft function may amplify a tubular injury biomarker signal (expressed as biomarker to UCr ratio), thereby increasing its clinical utility [15]. This was exactly the case with our results since we found significantly larger area under the ROC curve when concentrations of L-FABP (but also NGAL) were normalized to UCr. Interestingly, this improvement in ROC AUC was not found in previous study of NGAL [7], although the ROC AUC for NGAL was actually below that found in one of the previous studies where normalization to UCr was done (0.82 [7] vs. 0.9 [6]).

With such an important influence of UCr variation on the predictive ability of urinary biomarkers it is justified to inspect the predictive ability of UCr per se. In fact, we rigorously monitored diuresis and UCr concentration at predefined time periods after transplantation to examine the value of these traditional clinical parameters. Regarding diuresis, there was the largest difference between IGF and SGF/DGF groups in the first hours after transplantation and the difference gradually disappeared after 10 h time point (Figure 1). Our results clearly show that for diuresis the best predictive and discriminate values are found early after transplantation. Large urine output 4 h after transplantation had a very high positive predictive value for IGF (no patient developed SGF/DGF when urine output was above 328 ml/h 4 h post-transplant, see Table 3). The main disadvantage of using diuresis was that such a good urine output early on was present in only a minority of patients (17 out of 71 patients) and additional predictive markers are needed for the majority of patients with lower levels of diuresis.

To evaluate the UCr as predictor of allograft function we calculated UCr excretion rate, which is a product of urinary output and UCr concentration and as such may combine the predictive ability of both parameters. Excellent results were found. UCr excretion rate 10 hours post-transplant had numerically the largest area under ROC curve for discrimination between IGF and SGF/DGF groups (although not significantly different from L-FABP and NGAL). Our results show that at approximate cut-off of 0.5 mmol of UCr/h either 10 or 24 h post-transplant there is a very high positive and negative predictive value for early allograft function (89% positive and 91% negative predictive value 10 h post-transplant, see Table 3). Furthermore, when both traditional parameters were combined in the method of classification trees, little space for further improvement was left since this combination of markers adequately predicted first week outcome in 89% of cases. Further addition of L-FABP or NGAL did not improve the prediction significantly. According to our results, using diuresis and creatinine excretion rate is a cheap but accurate way to predict IGF or SGF/DGF as early as 10 h post-transplant.

The drawback of our study is a relatively low number of included patients. The number was however larger than in some of previous reports [6,16]. On the other hand the strength of the study is a rigorous protocol with predefined times for urine sampling after establishment of allograft reperfusion therefore eliminating the variability in timing of urine sampling. As this was a single centre study all patients were subjected to the same fluid management protocol and diuretic prescription practice eliminating the potential difference of these practices on postoperative diuresis.

## Conclusions

In conclusion, urinary L-FABP as a single predictive parameter demonstrated equivalent predictive accuracy for first week renal transplantation outcome to urinary NGAL. We found significantly improved predictive ability of both biomarkers when their urinary concentration was normalized to UCr. The results with L-FABP were best when sampled 48 h after establishment of allograft blood flow. Post-transplant diuresis is a valuable predictor of allograft function, but its ability to discriminate between IGF and SGF/DGF patients diminishes 10 h post-transplant. We have found that the predictive value of creatinine excretion rate (which combines creatinine urinary concentration and diuresis) was in fact numerically above all other tested biomarkers and statistically not inferior to other biomarkers, which is an original finding. We are certain that this results can be of help to clinicians, especially nowadays, when financial constraints may often prevent a wide accession to modern, but more expensive, urinary biomarkers. Further studies of urinary biomarkers should rigorously compare novel biomarkers to UCr excretion rate, due to its potential high predictive value, simplicity of measurement and low cost.

## Abbreviations

AKI: Acute kidney injury; DGF: Delayed graft function; NGAL: Neutrophil gelatinase-associated lipocalin; ROC: Receiver operating characteristic; L-FABP: Liver type fatty acid binding protein; UCr: Urinary creatinine; DDKT: Deceased-donor kidney transplant; SCr: Serum creatinine; IGF: Immediate graft function; SGF: Slow graft function.

## Competing interests

The authors declare no conflict of interest or competing interests in association with this work. The authors declare that the results presented in this paper have not been published elsewhere previously in whole or part, except in abstract format.

## Authors’ contributions

JP conceived the study, participated in research design, writing of the paper, performance of the research and data analysis. AŠ participated in research design, writing of the paper, performance of the research and data analysis. KŠ participated in research design, writing of the paper, performance of the research. AB participated in research design, writing of the paper, performance of the research. BL participated in research design and data analysis. MBP participated in research design and data analysis and in performance of research. JO contributed analytical tools, performed laboratory analyses and cooperated in drafting the manuscript. JL participated in revising the manuscript and data analysis. All authors read and approved the final manuscript.

## Pre-publication history

The pre-publication history for this paper can be accessed here:

http://www.biomedcentral.com/1471-2369/15/117/prepub

## Supplementary Material

Additional file 1 **UCr excretion rate for recipients without (A) and with residual urine output (B).** Solid line - IGF group, dashed line - SGF/DGF group. Bars represent 95% C.I. Click here for file

Additional file 2 **Classification trees for prediction of allograft function in the first post-transplant week.** Description of data: using the shown cut-off values the number of patients from each category (IGF or SGF/DGF) is shown. 4A - using urine output at 4 h post-transplant and UCr (urinary creatinine) excretion rate 10 h post-transplant; CHAID (chi-square automatic interaction detector) method for classification tree construction was used. 4B and 4C- using urinary L-FABP (48 h post-transplant) and NGAL (24 h post-transplant), both normalised to UCr; binary method for classification tree construction was used. Click here for file

## References

[B1] Siedlecki A Irish W Brennan DC Delayed graft function in the kidney transplant Am J Transplant 2011 11 2279 2296 10.1111/j.1600-6143.2011.03754.x 21929642 PMC3280444

[B2] Yarlagadda SG Coca SG Garg AX Doshi M Poggio E Marcus RJ Parikh CR Marked variation in the definition and diagnosis of delayed graft function: a systematic review Nephrol Dial Transplant 2008 23 2995 3003 10.1093/ndt/gfn158 18408075 PMC2727302

[B3] Yarlagadda SG Coca SG Formica RN Poggio ED Parikh CR Association between delayed graft function and allograft and patient survival: a systematic review and meta-analysis Nephrol Dial Transplant 2009 24 1039 1047 19103734 10.1093/ndt/gfn667

[B4] Irish WD Ilsley JN Schnitzler M a Feng S Brennan DC A risk prediction model for delayed graft function in the current era of deceased donor renal transplantation Am J Transplant 2010 10 2279 2286 10.1111/j.1600-6143.2010.03179.x 20883559

[B5] Schmidt-Ott KM Mori K Li JY Kalandadze A Cohen DJ Devarajan P Barasch J Dual action of neutrophil gelatinase-associated lipocalin J Am Soc Nephrol 2007 18 407 413 10.1681/ASN.2006080882 17229907

[B6] Parikh CR Jani A Mishra J Ma Q Kelly C Barasch J Edelstein CL Devarajan P Urine NGAL and IL-18 are predictive biomarkers for delayed graft function following kidney transplantation Am J Transplant 2006 6 1639 1645 10.1111/j.1600-6143.2006.01352.x 16827865

[B7] Hall IE Yarlagadda SG Coca SG Wang Z Doshi M Devarajan P Han WK Marcus RJ Parikh CR IL-18 and urinary NGAL predict dialysis and graft recovery after kidney transplantation J Am Soc Nephrol 2010 21 189 197 10.1681/ASN.2009030264 19762491 PMC2799276

[B8] Hollmen ME Kyllönen LE Inkinen KA Lalla MLT Salmela KT Urine neutrophil gelatinase-associated lipocalin is a marker of graft recovery after kidney transplantation Kidney Int 2011 79 89 98 10.1038/ki.2010.351 20861824

[B9] Yamamoto T Noiri E Ono Y Doi K Negishi K Kamijo A Kimura K Fujita T Kinukawa T Taniguchi H Nakamura K Goto M Shinozaki N Ohshima S Sugaya T Renal L-type fatty acid-binding protein in acute ischemic injury J Am Soc Nephrol 2007 18 2894 2902 10.1681/ASN.2007010097 17942962

[B10] Doi K Noiri E Sugaya T Urinary L-type fatty acid-binding protein as a new renal biomarker in critical care Curr Opin Crit Care 2010 16 545 549 10.1097/MCC.0b013e32833e2fa4 20736829

[B11] Przybylowski P Koc-Zorawska E Malyszko JS Kozlowska S Mysliwiec M Malyszko J Liver fatty-acid-binding protein in heart and kidney allograft recipients in relation to kidney function Transplant Proc 2011 43 3064 3067 10.1016/j.transproceed.2011.08.038 21996226

[B12] Matteucci E Carmellini M Bertoni C Boldrini E Mosca F Giampietro O Urinary excretion rates of multiple renal indicators after kidney transplantation: clinical significance for early graft outcome Ren Fail 1998 20 325 330 10.3109/08860229809045118 9574459

[B13] Kandus A Arnol M Bren AF Survey of renal transplantation in Slovenia Ther Apher Dial 2009 13 264 267 10.1111/j.1744-9987.2009.00721.x 19695056

[B14] Kohei J Ishida H Kazunari T Tsuchiya K Nitta K Neutrophil gelatinase-associated lipocalin is a sensitive biomarker for the early diagnosis of acute rejection after living-donor kidney transplantation Int Urol Nephrol 2013 45 1159 1167 10.1007/s11255-012-0321-y 23161375

[B15] Waikar SS Sabbisetti VS Bonventre JV Normalization of urinary biomarkers to creatinine during changes in glomerular filtration rate Kidney Int 2010 78 486 494 10.1038/ki.2010.165 20555318 PMC3025699

[B16] Nakamura T Sugaya T Node K Ueda Y Koide H Urinary excretion of liver-type fatty acid-binding protein in contrast medium-induced nephropathy Am J Kidney Dis 2006 47 439 444 10.1053/j.ajkd.2005.11.006 16490622

