# Supplementary figures and images for: Non-inferiority of creatinine excretion rate to urinary L-FABP and NGAL as predictors of early renal allograft function
Source: BMC Nephrol. 2014 Jul 16;15:117. doi: 10.1186/1471-2369-15-117 (PMC4107724; doi:10.1186/1471-2369-15-117)

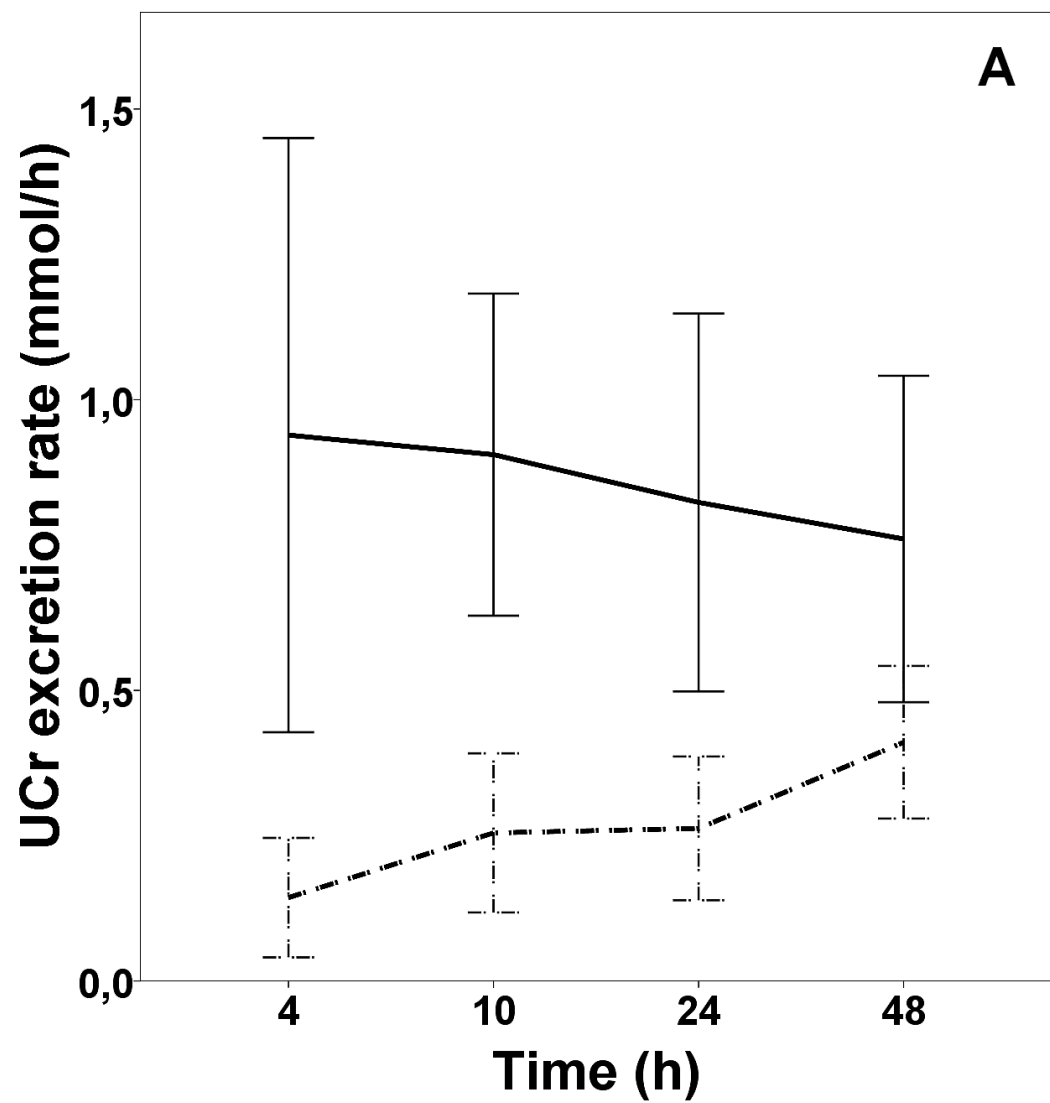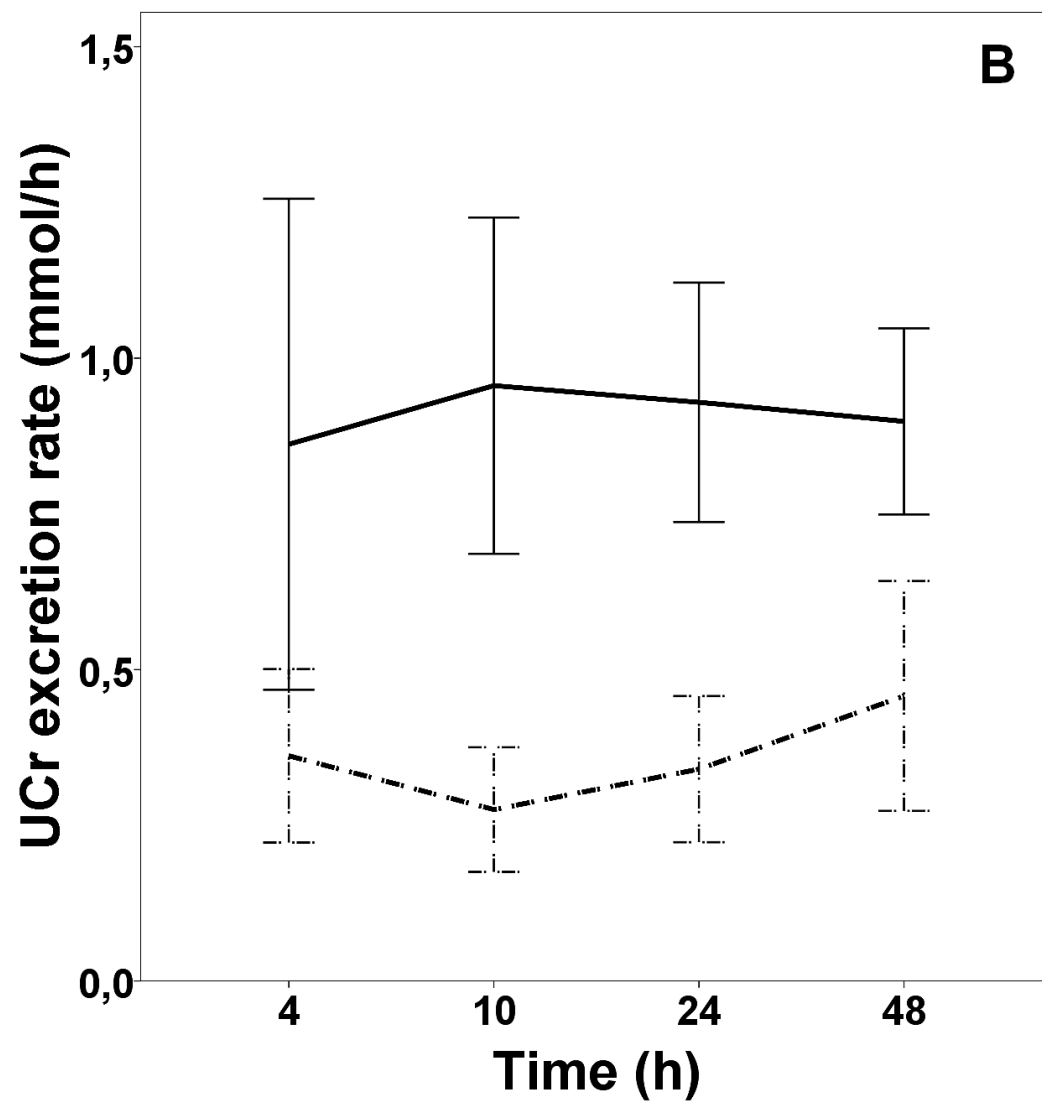

Supplement: Additional file 1 — UCr excretion rate for recipients without (A) and with residual urine output (B). Solid line - IGF group, dashed line - SGF/DGF group. Bars represent 95% C.I. [file 1471-2369-15-117-S1.pdf]

**A**

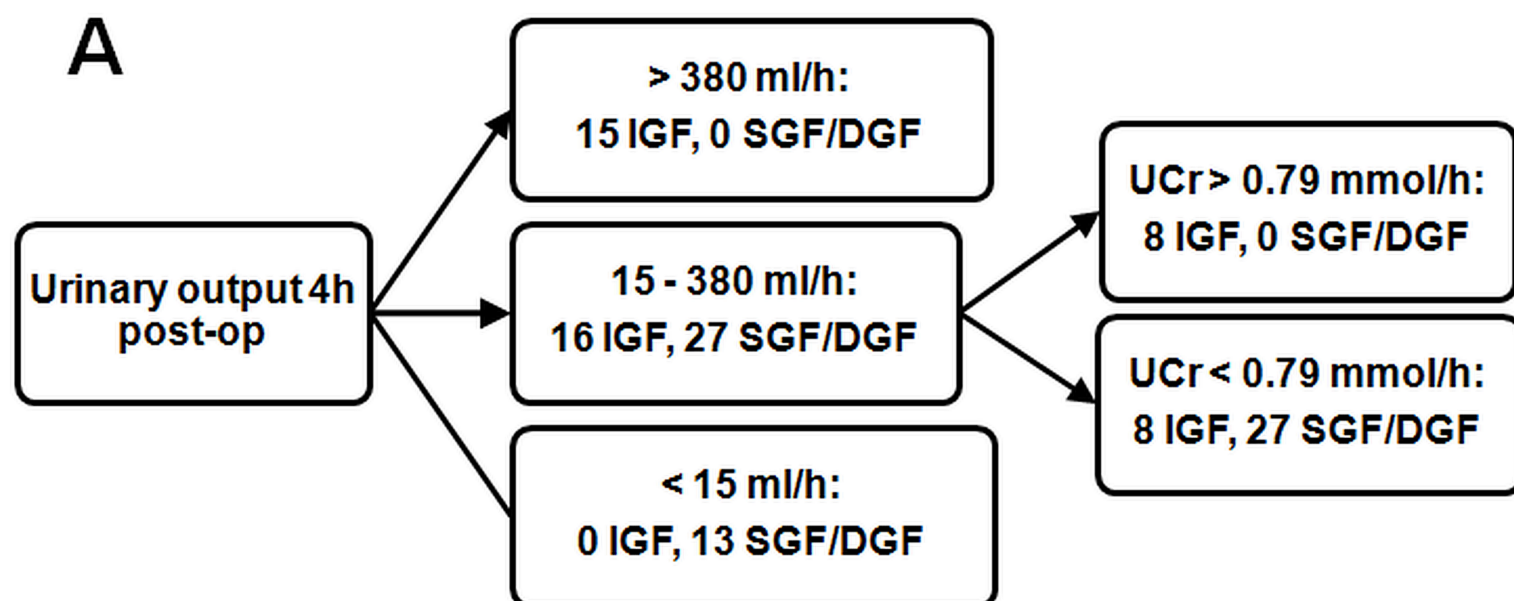

**B**

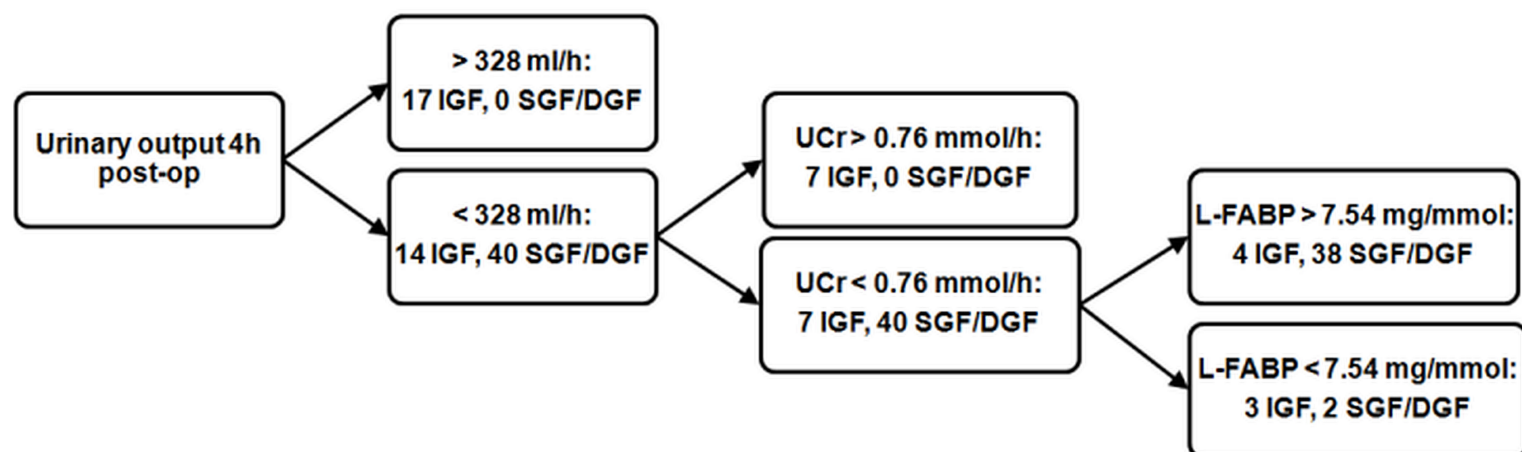

**C**

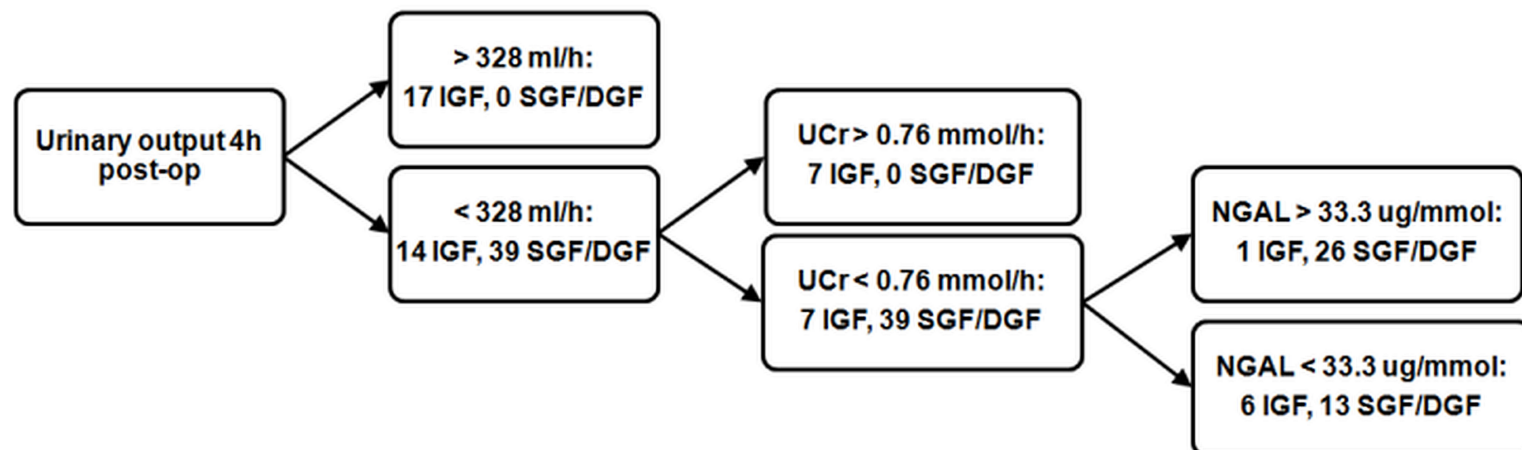

Supplement: Additional file 2 — Classification trees for prediction of allograft function in the first post-transplant week. Description of data: using the shown cut-off values the number of patients from each category (IGF or SGF/DGF) is shown. 4A - using urine output at 4 h post-transplant and UCr (urinary creatinine) excretion rate 10 h post-transplant; CHAID (chi-square automatic interaction detector) method for classification tree construction was used. 4B and 4C- using urinary L-FABP (48 h post-transplant) and NGAL (24 h post-transplant), both normalised to UCr; binary method for classification tree construction was used. [file 1471-2369-15-117-S2.pdf]
